# Intraoperative hemodialysis during open-heart surgery in patients with severe chronic kidney disease: a retrospective cohort study

**DOI:** 10.1186/s12882-023-03142-0

**Published:** 2023-03-30

**Authors:** Takahiro Inoue, Hiroshi Kuji, Kanako Nagaoka, Takafumi Akanuma, Junko Fukuda, Hiroki Matsui, Hiroaki Tanabe, Mamiko Ohara, Tomo Suzuki

**Affiliations:** 1 Department of Nephrology, Kameda Medical Center, 929, Higashicho, Kamogawa, Chiba 296-8602 Japan; 2 Department of Urology, Kameda Medical Center, Kamogawa, Japan; 3 Division of Nephrology and Hypertension, Department of Internal Medicine, St. Marianna University School of Medicine, Kanagawa, Japan; 4 Department of Clinical Epidemiology and Health Economics, School of Public Health, The University of Tokyo, Tokyo, Japan; 5 Department of Cardiovascular Surgery, Kameda Medical Center, Kamogawa, Japan

**Keywords:** Intraoperative hemodialysis, Cardiac surgery, Chronic kidney disease, Acute kidney injury

## Abstract

**Background:**

Acute kidney injury and chronic kidney disease (CKD) after cardiac surgery are associated with poor renal prognosis and increased mortality. The impact of intraoperative hemodialysis (IHD) on postoperative renal function remains unknown. We aimed to evaluate the utility of IHD during open-heart surgery in patients with severe non-dialysis-dependent chronic kidney disease (CKD-NDD) and its association with clinical outcomes.

**Methods:**

This was a single-center retrospective cohort study that employed IHD during non-emergency open-heart surgery in patients with CKD stage G4 or G5. Patients who underwent emergent surgery, chronic dialysis, and/or kidney transplantation were excluded. We retrospectively compared the clinical characteristics and outcomes between patients from the IHD and non-IHD groups. The primary outcomes were 90-day mortality and postoperative initiation of renal replacement therapy (RRT).

**Results:**

Twenty-eight patients were categorized into the IHD group and 33 into the non-IHD group. When comparing the IHD and non-IHD groups, men accounted for 60.7 vs. 50.3% of patients, the mean patient age was 74.5 (standard deviation [SD] 7.0) vs. 72.9 (SD 9.4) years (*p* = 0.744), and the proportion of patients with CKD G4 was 67.9 vs. 84.9% (*p* = 0.138). Regarding clinical outcomes, no significant differences were observed in the 90-day mortality (7.1 vs. 3.0%; *p* = 0.482) and 30-day RRT (17.9 vs. 30.3%; *p* = 0.373) rates between the groups. Among the patients with CKD G4, the IHD group had significantly lower 30-day RRT rates than the non-IHD group (0 vs. 25.0%; *p* = 0.032). RRT initiation was less likely for patients with CKD G4 (odds ratio 0.07, 95% confidence interval [CI] 0.01–0.37; *p* = 0.002); however, IHD did not significantly decrease the incidence of poor clinical outcomes (odds ratio 0.20, 95% CI 0.04–1.07; *p* = 0.061).

**Conclusions:**

IHD during open-heart surgery in patients with CKD-NDD did not improve their clinical outcomes with regards to postoperative dialysis. However, for patients with CKD G4, IHD may be useful for postoperative cardiac management.

## Background

Acute kidney injury (AKI) is a common and serious postoperative complication of cardiac surgery. Nephrotoxins, metabolic abnormalities, ischemia and reperfusion injury, pre-existing chronic diseases, inflammation, and oxidative stress may lead to the postoperative AKI development [1]. Patients with AKI have higher rates of short-term and long-term mortality, prolonged duration of hospital stay, and higher hospital costs [2]. Among patients with AKI after cardiac surgery, 1–5% require renal replacement therapy (RRT) for severe postoperative AKI, and a strikingly high in-hospital mortality rate exceeding 40% has been reported in these patients [3, 4]. Currently, there is no standardized treatment for AKI. Therefore, prevention and risk-factor management are important for postoperative care after cardiac surgery.

RRT is frequently required to treat severe AKI-associated hyperkalemia, fluid retention, uremia, and metabolic acidosis. Although the indications for RRT have been extensively studied, the most appropriate criteria for and timing of cardiac surgery have not been definitively established. A few randomized controlled trials suggest that early or preemptive RRT in patients with non-dialysis-dependent chronic kidney disease (CKD-NDD) undergoing cardiac surgery is associated with a lower mortality rate and shorter stay in the intensive care unit (ICU) [5–8]. Additionally, Michell et al. reported that intraoperative hemodialysis (IHD) is safe and prevents hyperkalemia in patients with CKD-NDD undergoing cardiac surgery [9]. Therefore, at our institution, IHD was concomitantly performed for patients with CKD-NDD and dialysis-dependent CKD to ameliorate fluid overload, acidosis, and electrolyte abnormalities. However, studies reporting the use of IHD in patients with CKD are scarce.

This study aimed to elucidate the association between IHD and clinical outcomes of patients with CKD-NDD undergoing open-heart surgery. We hypothesized that IHD may contribute to the reduction in mortality rate and ameliorate the deterioration of kidney function in these patients.

## Methods

### Study population

**Fig. 1:**
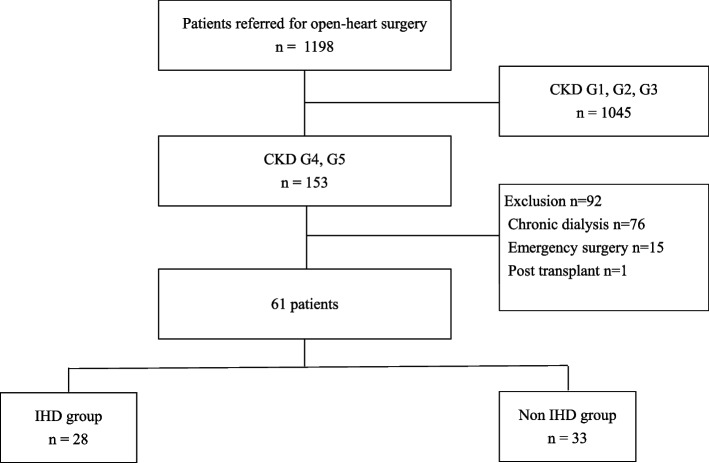
Study flowchart

This was a single-center retrospective cohort study that aimed to evaluate the association between IHD and the clinical outcomes of patients with CKD-NDD who underwent non-emergency open-heart surgery between January 2008 and December 2018. All data were collected at the Kameda Medical Center. We instituted IHD for patients with chronic kidney disease (CKD), particularly for those undergoing chronic hemodialysis, in September 2013 following the appointment of a new Chief in the department of cardiovascular surgery. All patients with dialysis-dependent CKD underwent IHD, whereas IHD use for patients with CKD-NDD was determined on a case-by-case basis after consultation between the cardiac surgeon and nephrologist. Figure 1 depicts a flowchart representing patient enrollment and group stratification. Patients who underwent emergent surgery, chronic dialysis, and/or kidney transplantation were excluded from the study.

### Intraoperative hemodialysis

After the induction of anesthesia, a temporary vascular catheter was placed in the femoral or internal jugular vein by a cardiac surgeon concurrent with the initiation of thoracotomy. IHD was immediately started using a hemodialysis machine (DBB-100NS or DCS-100NS; Nikkiso., Ltd., Japan) and terminated once the chest was closed. The duration of an IHD session was approximately 4–5 h. The dialyzer used was an ethylene vinyl alcohol membrane with a surface area of 1.5 m^2^. The dialysate contained 140, 2.0, 1.375, and 27.5 mmol/L of sodium, potassium, calcium, and bicarbonate, respectively. The blood flow through the dialyzer was maintained at a flow rate of 150 mL/min and the dialysate at 500 mL/min. Additionally, nafamostat mesylate, which is an anticoagulant and a synthetic serine protease inhibitor, was continuously injected at a rate of 30 mg/h. The target serum potassium level was 4.0 mEq/L.

### Study outcomes

The medical records of the patients who underwent cardiac surgery were retrospectively reviewed. We compared the IHD group after September 2013 with the non-IHD group from before September 2013. The primary outcomes were the 90-day mortality and postoperative RRT initiation rates. A nephrologist decided whether RRT should be initiated after cardiac surgery based on the electrolyte abnormalities, uremia, acidosis, and fluid overload in the patient. The secondary outcomes were the durations of hospital stay and postoperative intubation and RRT continuation after discharge. Furthermore, we examined intraoperative parameters, including blood transfusion volume, type of surgery, and use of intra-aortic balloon pumping. For the evaluation of renal function, estimated glomerular filtration rate (eGFR) (mL/min/1.73 m^2^) was calculated according to serum creatinine, sex, and age [10].

### Statistical analyses

Continuous and categorical variables are presented as mean ± standard deviation. Demographic variables were compared between the groups using an independent t-test, and Fisher’s exact test was used to analyze the time to morbidity onset. Multivariable logistic regression analysis predicting the odds of RRT initiation was performed by including IHD and the proportion of patients with CKD stage G4 (CKD G4). Statistical significance was set at *p* < 0.05. Statistical analyses were performed using the JMP software, version 11.0.0 (SAS Institute Inc., Cary, NC, USA).

## Results

### Participants

Of 1,198 patients who underwent open-heart surgery at our institution, 153 had CKD G4 or G5 among whom, 92 were excluded because they underwent chronic dialysis (*n* = 76), emergent surgery (*n* = 15), or renal transplantation (*n* = 1). Ultimately, 61 patients were included in the study and categorized into the IHD (28 patients) or non-IHD (33 patients) group. Among the patients with CKD G3b, 9 patients were on IHD, excluding those who underwent emergency surgery and renal transplantation. Basic demographic data and pre-, and postoperative characteristics of patients from both groups are summarized in Table 1. When comparing the IHD and non-IHD groups, men accounted for 60.7 vs. 50.3% of patients, the age of the patients was 74.5 ± 7.0 vs. 72.9 ± 9.4 years (*p* = 0.339), and the proportion of patients with CKD G4 was 67.9 vs. 84.9% and that of those with CKD G5 was 32.1 vs. 15.2%, respectively. Furthermore, preoperative eGFR in the IHD group tended to be lower than that in the non-IHD group, although not significantly so (19.5 ± 5.9 vs. 22.2 ± 5.2 mL/min/1.73 m^2^; *p* = 0.056). In addition, the proportion of patients with diabetes mellitus was 35.7 vs. 42.4% (*p* = 0.384). Thus, the baseline characteristics did not significantly differ between the groups.

**Table 1:** Demographic characteristics and pre-, intra-, and postoperative data of study participants

	IHD (*N* = 28)	Non-IHD (*N* = 33)	*p*-value
Male, n (%)	18 (60.7)	16 (50.3)	0.744
Age, years (SD)	74.5 (7.0)	72.9 ± 9.4	0.339
HTN, n (%)	25 (89.2)	31 (93.9)	0.653
DM, n (%)	10 (35.7)	14 (42.4)	0.611
CVD, n (%)	5 (17.9)	8 (24.2)	0.540
Peripheral arterial disease, n (%)	1 (3.6)	3 (9.1)	0.618
Serum creatinine, mg/dL (SD)	2.7 (1.1)	2.3 (1.1)	0.131
eGFR, mL/min/1.73 m^2^ (SD)	19.5 (5.9)	22.2 (5.2)	0.056
CKD G4, n (%)	19 (67.9)	28 (84.9)	0.138
CKD G5, n (%)	9 (32.1)	5 (15.2)	
BUN, mg/dL (SD)	41.5 (18.6)	40.4 (16.7)	0.811
Serum albumin, g/dL (SD)	3.3 (0.7)	3.3 (0.6)	0.988
Serum potassium, mEq/L (SD)	4.5 (0.4)	4.4 (0.6)	0.435
Preoperative medication, n (%)			
β-blocker	15 (53.6)	7 (21.2)	0.073
Statin	18 (64.3)	21 (63.6)	0.980
RAS-inhibitor	15 (53.6)	13 (39.4)	0.501
Preoperative Hb, g/dL (SD)	10.8 (1.6)	11.0 (1.5)	0.587
Postoperative Hb, g/dL (SD)	10.8 (1.2)	10.0 (1.0)	0.005
Preoperative EF, % (SD)	55.2 (12.8)	56.5 (14.0)	0.708
Blood transfusion during operation, mL (SD)	1330 (709)	1324 (860)	0.975
Type of surgery			
CABG, n	8	16	0.291
CABG + valve operation, n	4	2	
AVR, n	5	7	
MVR, n	1	4	
MVP, n	5	1	
TAA repair, n	2	1	
Other procedures, n	3	2	
IABP, n (%)	1 (3.6)	4 (12.1)	0.262
Perioperative medications			
Inotropic agents, n (%)	12 (42.9)	14 (42.4)	0.983
Diuretics, n (%)	25 (89.3)	22 (66.7)	0.452
Glucocorticoids, n (%)	3 (10.7)	1 (3.0)	0.259
Carperitide, n (%)	5 (17.9)	9 (27.3)	0.489

*CKD* Chronic kidney disease, *eGFR* Estimated glomerular filtration rate, *SD* Standard deviation, *BUN* Blood urea nitrogen, *RAS* Renin-angiotensin system, *IHD* Intraoperative hemodialysis, *HTN* Hypertension, *DM* Diabetes mellitus, *CVD* Cardiovascular disease, *Hb* Hemoglobin, *EF* Ejection fraction, *CABG* Coronary artery bypass grafting, *AVR* Aortic valve replacement, *MVR* Mitral valve replacement, *MVP* Mitral valvuloplasty, *TAA* thoracic aortic aneurysm, *IABP* Inta-aortic balloon pumping

### Intraoperative and postoperative characteristics

Intraoperative and postoperative patient parameters are presented in Table 1. Among 61 patients, 23 (37.7%), 24 (39.3%), 6 (9.8%), 3 (4.9%), and 5 (8.2%) patients underwent isolated heart valve, isolated coronary artery bypass grafting, both valve and coronary artery bypass grafting, thoracic aortic aneurysm repair, and other open-heart surgeries, respectively. Intraoperative variables, including the type of surgery, requirement of an intra-aortic balloon pump, and perioperative medications, such as inotropic agents, diuretics, glucocorticoids, and carperitide, were not significantly different between the two groups. The postoperative hemoglobin (Hb) level was significantly higher in patients from the IHD group than that in those from the non-IHD group (10.8 ± 1.2 vs. 10.0 ± 1.0 g/dL; *p* = 0.005); however, the blood transfusion volumes between the groups did not significantly differ (1330 ± 709 vs. 1324 ± 860 mL; *p* = 0.975).

### Clinical outcomes

Clinical outcomes are shown in Table 2. No significant differences were observed in the 90-day mortality rate (7.1 vs. 3.0%; *p* = 0.482), 30-day RRT rate (17.9 vs. 30.3%; *p* = 0.373), duration of hospital stay (32.2 ± 30.2 vs. 34.2 ± 36.3 days; *p* = 0.833), and duration of postoperative intubation (4.2 ± 8.65 vs. 2.68 ± 2.90 days; *p* = 0.379) between the IHD and non-IHD groups, respectively. However, among patients with CKD G4 (all of whom presented with fluid overload), the rate of 30-day RRT initiation was significantly lower in the IHD group than in the non-IHD group (0 vs. 25.0%; *p* = 0.032). In contrast, the 30-day RRT rate in patients with CKD G5 was not significantly different between the two groups (55.6 vs. 60.0%; *p* = 1.000). After discharge, two patients from the IHD group and two from the non-IHD group continued receiving hemodialysis, all four with CKD G5.

**Table 2:** Clinical outcomes of study participants after cardiac surgery

	IHD (*N* = 28)	Non-IHD (*N* = 33)	*p*-value
90-day mortality, n (%)	2 (7.1)	0 (0)	0.207
Inpatient hospitalization, days (SD)	23.7 (18.7)	35.1 (81.4)	0.474
Duration of postoperative intubation, days (SD)	3.5 (7.1)	2.8 (3.1)	0.587
30-day RRT initiation, n (%)	5 (17. 9)	10 (30.3)	0.373
CKD G4, n (%)	0 (0.0)	7 (25.0)	0.032
CKD G5, n (%)	5 (55.6)	3 (60.0)	1.000
Causes of postoperative RRT initiation, n			
Acidosis			
CKD G4	0	0	
CKD G5	1	0	
Uremia			
CKD G4	0	0	
CKD G5	0	1	
Electrolyte abnormalities			
CKD G4	0	0	
CKD G5	1	0	
Fluid overload			
CKD G4	3	7	
CKD G5	0	2	
Continuation of RRT after discharge	2	2	
CKD G4, n (%)	0 (0)	0 (0)	
CKD G5, n (%)	2 (100)	2 (100)	

*IHD* Intraoperative hemodialysis, *SD* Standard deviation, *RRT* Renal replacement therapy, *CKD* Chronic kidney disease

In the multivariate model, which included the rates of incidence of CKD G4 and IHD, patients with CKD G4 had a lower likelihood of RRT initiation (odds ratio 0.07, 95% confidence interval [CI] 0.01–0.37; *p* = 0.002); however, IHD did not significantly lower the rate of RRT initiation (odds ratio 0.20, 95% CI 0.04–1.07; *p* = 0.061) (Table 3).

**Table 3:** Odds of RRT initiation in the IHD and non-IHD groups

	Odds ratio (95% CI)	*p*-value
IHD	0.20 (0.04–1.07)	0.061
CKD G4	0.07 (0.01–0.37)	0.002

*RRT* Renal replacement therapy, *CI* Confidence interval, *IHD* Intraoperative hemodialysis, *CKD* Chronic kidney disease

## Discussion

In the present study, we retrospectively evaluated the clinical efficacy of IHD in patients with CKD-NDD who underwent cardiac surgery. No significant differences were observed between the outcomes of the IHD and non-IHD groups. The rate of postoperative RRT initiation in patients with CKD G4 was significantly lower in the IHD group than that in the non-IHD group. However, IHD did not significantly decrease the incidence of clinical outcomes in the multivariate model.

The development of AKI after cardiac surgery (particularly after cardiopulmonary bypass surgery) is associated with prolonged ICU and hospital stays and an increased risk of death [11–15]. Many patients develop multiple organ failure, thereby requiring assisted ventilation, intra-aortic balloon counterpulsation, continuous inotropic medication, and occasionally, extracorporeal life support. Among patients who required hemodialysis in recent reports, 64% required permanent dialysis and approximately 90% died within 1 year [16, 17]. Additionally, patients with postoperative AKI may require maintenance hemodialysis. Therefore, the early treatment of severe postoperative AKI, particularly after cardiac surgery, is important.

Durmaz et al. reported that perioperative prophylactic RRT decreased both operative mortality and morbidity in high-risk patients with CKD [18]. Sugahara et al. further reported that early initiation of RRT improved the survival of patients with AKI following cardiac surgery [19]. Thus, prophylactic or early RRT may be useful in patients with CKD-NDD after cardiac surgery.

Multiple strategies are commonly employed to improve intraoperative AKI. The use of dopamine, furosemide, fenoldopam, and human atrial natriuretic peptides prevents AKI [20, 21]. In the present study, perioperative drug therapy, type of surgery, and patient characteristics did not differ significantly between the two groups. Furthermore, red blood cell transfusion is independently associated with the incidence of AKI after cardiac surgery [22, 23]. In our study, the blood transfusion volume did not differ between the IHD and non-IHD groups.

Additionally, fluid overload was the most frequent cause of RRT initiation in our study. Fluid overload is reportedly a risk factor for mortality and can prolong the postoperative ventilation duration [24, 25]. Several studies have shown that hemofiltration improves cardiorespiratory function in patients with AKI and cardiac shock after surgery [6, 26] and metabolic acidosis reduces the cardiac output [27]. In our study, the postoperative Hb levels were significantly higher in the IHD group than in the non-IHD group despite no differences in the blood transfusion volume and preoperative Hb levels between the groups. Therefore, the non-IHD group may have had more fluid retention than the IHD group. IHD may lead to better control of fluid status and improve uremia and acidosis.

To the best of our knowledge, studies investigating IHD in patients with CKD-NDD who undergo cardiac surgery are rare. During liver transplantation in patients with CKD-NDD, IHD ameliorates fluid overload, acidosis, and electrolyte abnormalities, and is reportedly safe and effective [28]. IHD may be helpful in achieving an even or negative fluid balance with minimal hemodynamic changes during surgery, despite the administration of significant amounts of blood products and crystalloids.

Our study had a few limitations. First, this was a retrospective observational study conducted at a single center, and the sample size was small. Second, the medical and nursing staff in charge of the IHD and non-IHD groups differed. In particular, the main surgeon for patients from the IHD and non-IHD groups was different, which may have influenced the clinical outcomes of both groups. Third, postoperative dialysis was initiated at the discretion of the nephrologist. However, fluid overload was the major cause of dialysis. Therefore, the impact of the decision by the nephrologist on the clinical outcomes in this study was minimal. Fourth, the indications for IHD in patients with CKD-NDD at our institution were unclear. In our cohort, all patients with CKD stages G4 and G5 underwent IHD, but only 9 patients were in CKD stage G3b. This was probably a low percentage of all patients with CKD G3b patients (data not shown). Fifth, the perioperative infusion volume was unknown; therefore, anesthesiologists might have used different infusion strategies for both groups during the long study period. Future studies should be multi-centered ones or include of varied ethnicities.

## Conclusions

IHD during open-heart surgery in patients with CKD-NDD did not improve their clinical outcomes in terms of the need for postoperative dialysis. However, IHD may be useful for the postoperative management of patients with CKD G4, especially in cases of fluid overload. Further studies, including a randomized control trial and with larger sample sizes, are needed to elucidate the safety and effectiveness of IHD use in actual clinical practice.

## Data Availability

The datasets used and/or analyzed during the current study are available from the corresponding author upon reasonable request.

## Abbreviations

AKI: Acute kidney injury
CI: Confidence interval
CKD: Chronic kidney disease
CKD-NDD: Non-dialysis-dependent chronic kidney disease
eGFR: Estimated glomerular filtration rate
Hb: Hemoglobin
IHD: Intraoperative hemodialysis
ICU: Intensive care unit
RRT: Renal replacement therapy

## References

[1] Bellomo R, Auriemma S, Fabbri A, D’Onofrio A, Katz N, McCullough PA, et al. The pathophysiology of cardiac surgery-associated acute kidney injury (CSA-AKI). Int J Artif Organs. 2008;31:166–78. 10.1177/039139880803100210 18311733

[2] Chertow GM, Burdick E, Honour M, Bonventre JV, Bates DW (2005). Acute kidney injury, mortality, length of stay, and costs in hospitalized patients. J Am Soc Nephrol.

[3] Parikh CR, Devarajan P, Zappitelli M, Sint K, Thiessen-Philbrook H, Li S (2011). Postoperative biomarkers predict acute kidney injury and poor outcomes after pediatric cardiac surgery. J Am Soc Nephrol.

[4] Mariscalco G, Lorusso R, Dominici C, Renzulli A, Sala A (2011). Acute kidney injury: a relevant complication after cardiac surgery. Ann Thorac Surg.

[5] Sun S, Ma F, Li Q, Bai M, Li Y, Yu Y (2017). Risk model for deaths and renal replacement therapy dependence in patients with acute kidney injury after cardiac surgery. Interact Cardiovasc Thorac Surg.

[6] Elahi MM, Lim MY, Joseph RN, Dhannapuneni RRV, Spyt TJ (2004). Early hemofiltration improves survival in post-cardiotomy patients with acute renal failure. Eur J Cardiothorac Surg.

[7] Zou H, Hong Q, Xu G (2017). Early versus late initiation of renal replacement therapy impacts mortality in patients with acute kidney injury post cardiac surgery: a meta-analysis. Crit Care..

[8] Bojan M, Gioanni S, Vouhé PR, Journois D, Pouard P (2012). Early initiation of peritoneal dialysis in neonates and infants with acute kidney injury following cardiac surgery is associated with a significant decrease in mortality. Kidney Int.

[9] Khoo MSC, Braden GL, Deaton D, Owen S, Germain M, O’Shea M, et al. Outcome and complications of intraoperative hemodialysis during cardiopulmonary bypass with potassium-rich cardioplegia. Am J Kidney Dis. 2003;41:1247–56. 10.1016/s0272-6386(03)00369-x 12776278

[10] Matsuo S, Imai E, Horio M, Yasuda Y, Tomita K, Nitta K (2009). Revised equations for estimated GFR from serum creatinine in Japan. Am J Kidney Dis.

[11] O’Neal JB, Shaw AD, Billings FT 4th. Acute kidney injury following cardiac surgery: current understanding and future directions. Crit Care. 2016;20:187. 10.1186/s13054-016-1352-z PMC4931708 27373799

[12] Thiele RH, Isbell JM, Rosner MH (2015). AKI associated with cardiac surgery. Clin J Am Soc Nephrol.

[13] Kuitunen A, Vento A, Suojaranta-Ylinen R, Pettilä V (2006). Acute renal failure after cardiac surgery: evaluation of the RIFLE classification. Ann Thorac Surg.

[14] Robert AM, Kramer RS, Dacey LJ, Charlesworth DC, Leavitt BJ, Helm RE (2010). Cardiac surgery-associated acute kidney injury: a comparison of two consensus criteria. Ann Thorac Surg.

[15] Haase M, Bellomo R, Matalanis G, Calzavacca P, Dragun D, Haase-Fielitz A (2009). A comparison of the RIFLE and Acute Kidney Injury Network classifications for cardiac surgery-associated acute kidney injury: a prospective cohort study. J Thorac Cardiovasc Surg.

[16] Leacche M, Rawn JD, Mihaljevic T, Lin J, Karavas AN, Paul S (2004). Outcomes in patients with normal serum creatinine and with artificial renal support for acute renal failure developing after coronary artery bypass grafting. Am J Cardiol.

[17] Thakar CV, Liangos O, Yared JP, Nelson DA, Hariachar S, Paganini EP (2003). Predicting acute renal failure after cardiac surgery: validation and re-definition of a risk-stratification algorithm. Hemodial Int.

[18] Durmaz I, Yagdi T, Calkavur T, Mahmudov R, Apaydin AZ, Posacioglu H (2003). Prophylactic dialysis in patients with renal dysfunction undergoing on-pump coronary artery bypass surgery. Ann Thorac Surg.

[19] Sugahara S, Suzuki H (2004). Early start on continuous hemodialysis therapy improves survival rate in patients with acute renal failure following coronary bypass surgery. Hemodial Int.

[20] Sirivella S, Gielchinsky I, Parsonnet V (2000). Mannitol, furosemide, and dopamine infusion in postoperative renal failure complicating cardiac surgery. Ann Thorac Surg.

[21] Sezai A, Hata M, Niino T, Yoshitake I, Unosawa S, Wakui S (2011). Results of low-dose human atrial natriuretic peptide infusion in nondialysis patients with chronic kidney disease undergoing coronary artery bypass grafting: the NU-HIT (Nihon University working group study of low-dose HANP Infusion Therapy during cardiac surgery) trial for CKD. J Am Coll Cardiol.

[22] Khan UA, Coca SG, Hong K, Koyner JL, Garg AX, Passik CS (2014). Blood transfusions are associated with urinary biomarkers of kidney injury in cardiac surgery. J Thorac Cardiovasc Surg.

[23] Karkouti K, Grocott HP, Hall R, Jessen ME, Kruger C, Lerner AB (2015). Interrelationship of preoperative anemia, intraoperative anemia, and red blood cell transfusion as potentially modifiable risk factors for acute kidney injury in cardiac surgery: a historical multicentre cohort study. Can J Anaesth.

[24] Shim HJ, Jang JY, Lee SH, Lee JG (2014). The effect of positive balance on the outcomes of critically ill noncardiac postsurgical patients: a retrospective cohort study. J Crit Care.

[25] Li C, Wang H, Liu N, Jia M, Zhang H, Xi X (2018). Early negative fluid balance is associated with lower mortality after cardiovascular surgery. Perfusion.

[26] Coraim FI, Wolner E (1995). Continuous hemofiltration for the failing heart. New Horiz.

[27] Naka T, Bellomo R (2004). Bench-to-bedside review: treating acid-base abnormalities in the intensive care unit-the role of renal replacement therapy. Crit Care.

[28] Nadim MK, Annanthapanyasut W, Matsuoka L, Appachu K, Boyajian M, Ji L (2014). Intraoperative hemodialysis during liver transplantation: a decade of experience. Liver Transpl.

